# Organ Preservation and Late Functional Outcome in Oropharyngeal Carcinoma: Rationale of EORTC 1420, the “Best of” Trial

**DOI:** 10.3389/fonc.2019.00999

**Published:** 2019-10-22

**Authors:** Jean-Jacques Stelmes, Vincent Gregoire, Vincent Vander Poorten, Wojciech Golusiñski, Mateusz Szewczyk, Terry Jones, Mohssen Ansarin, Martina A. Broglie, Roland Giger, Jens Peter Klussmann, Mererid Evans, Jean Bourhis, C. René Leemans, Giuseppe Spriano, Andreas Dietz, Keith Hunter, Frank Zimmermann, Ingeborg Tinhofer, Joanne M. Patterson, Silvana Quaglini, Anne-Sophie Govaerts, Catherine Fortpied, Christian Simon

**Affiliations:** ^1^Department of Radiation Oncology, University Hospital Zurich (USZ), Zurich, Switzerland; ^2^Department of Radiation Oncology, Centre Léon Bérard, Lyon, France; ^3^Section Head and Neck Oncology, Department of Oncology, KU Leuven, Leuven, Belgium; ^4^Department of Head and Neck Surgery, The Greater Poland Cancer Centre, Poznan University of Medical Sciences, Poznań, Poland; ^5^Liverpool Head and Neck Centre, University of Liverpool, Liverpool, United Kingdom; ^6^Division of Otolaryngology Head and Neck Surgery, European Institute of Oncology, Milan, Italy; ^7^Department of Otolaryngology-Head and Neck Surgery, University Hospital Zurich (USZ), Zurich, Switzerland; ^8^Department of Oto-Rhino-Laryngology, Head and Neck Surgery, Bern University Hospital and University of Bern, Inselspital, Bern, Switzerland; ^9^Department of Otolaryngology-Head and Neck Surgery, Medical Faculty, University of Cologne, Cologne, Germany; ^10^Velindre University NHS Trust, Wales, United Kingdom; ^11^Department of Radiation Oncology, CHUV, Lausanne, Switzerland; ^12^Department of Otolaryngology-Head and Neck Surgery, Cancer Center Amsterdam, Amsterdam University Medical Centres, VU University, Amsterdam, Netherlands; ^13^Department of Otolaryngology Head and Neck Surgery, Humanitas University Milan, Rozzano, Italy; ^14^Department of Otolaryngology, Head and Neck Surgery, University Leipzig, Leipzig, Germany; ^15^Academic Unit of Oral Medicine, Pathology and Surgery, School of Clinical Dentistry, University of Sheffield, Sheffield, United Kingdom; ^16^Department of Radiation Oncology, University of Basel, Basel, Switzerland; ^17^Charité – Universitätsmedizin Berlin, Corporate Member of Freie Universität Berlin, Humboldt-Universität zu Berlin, Berlin Institute of Health, Department of Radiooncology and Radiotherapy, Berlin, Germany; ^18^Institute for Health and Society, Newcastle University, Newcastle upon Tyne, United Kingdom; ^19^Department of Electrical, Computer and Biomedical Engineering, University of Pavia, Pavia, Italy; ^20^EORTC Headquarters, Brussels, Belgium; ^21^Service d'Oto-Rhino-Laryngologie – Chirurgie Cervico-Faciale, Centre Hospitalier Universitaire Vaudois (CHUV), Université de Lausanne (UNIL), Lausanne, Switzerland

**Keywords:** head and neck cancer, oropharyngeal cancer, organ preservation, EORTC 1420, functional outcome

## Abstract

Dysphagia represents one of the most serious adverse events after curative-intent treatments with a tremendous impact on quality of life in patients with head and neck cancers. Novel surgical and radiation therapy techniques have been developed to better preserve swallowing function, while not negatively influencing local control and/or overall survival. This review focuses on the current literature of swallowing outcomes after curative treatment strategies. Available results from recent studies relevant to this topic are presented, demonstrating the potential role of new treatment modalities for early- and intermediate-stage oropharyngeal cancers. Based on this, we present the rationale and design of the currently active EORTC 1420 “Best of” trial, and highlight the potential of this study to help prioritizing either surgery- or radiation-based treatment modalities for the treatment of oropharyngeal cancer in the future.

## Introduction

The ideal treatment for any cancer should control the disease, but inflict little, if any, toxicity. This paradigm is even more important for cancers with a good prognosis, as patients who survive for a longer period of time will have to deal with a full spectrum of late-toxic events.

Head and neck cancers (HNC) and their treatment are inevitably leading to dysphagia, which profoundly affects quality of life (QOL). Dysphagia is the result of a dysfunction of swallowing, one of the most complex physiological processes coordinated by 30 different muscles and 6 cranial nerves (1, 2). In early-stage HNC patients, among other stages, this is directly linked to social and psychological problems (3).

Novel surgical- and radiotherapy (RT)-based strategies have been developed within the past 10–20 years that allow for a more targeted approach to the cure of early- and intermediate-stage oropharyngeal cancers (OPSCC). In surgery, transoral surgical endoscopic techniques (TOS) emerged, allowing for a reduction of access trauma. This helped improve the functional recovery of patients, particularly in terms of acute and late dysphagia (4). For RT, the emergence of static and dynamic Intensity Modulated Radiation Therapy (IMRT, VMAT) allowed a better sparing of healthy tissues surrounding the cancer and thus also led to better functional recovery (4).

In this review, we address the rationale and design of EORTC 1420 “Best-of,” a trial comparing transoral surgery with intensity modified radiation therapy (IMRT) and highlight how this currently active phase III study could potentially change the therapeutic landscape in this patient population and beyond for HNC in general.

Although there is no prior prospective clinical trial that has compared surgery vs. RT for early-stage and intermediate-stage OPSCC in terms of survival, many retrospective studies have shown equivalent disease-specific survival between these two treatment modalities (DSS) rates (5–7). The largest and most recently reported data from a multi-center US retrospective study on approximately 400 patients with early and moderately advanced OPSCC [89% OPSCC and 6% supraglottic cancers (SGSCC)] treated with transoral robotic surgery (TORS) reported a DSS of 94.5% at 2 years (6). Previous literature reviews provide similar outcome results in terms of DSS at 2 years of between 86 and 95% (8).

In an observational cohort study of 42 patients treated between 1999 and 2001 for T1-2 N0 M0 oropharyngeal cancers, patients were divided into those treated with TOS (+/– adjuvant RT) and nonsurgical treatment with RT or CRT. Five-year DSS was 69 vs. 60%, respectively (*p* = 0.22). The authors concluded that surgical treatment allows for accurate staging and avoidance of RT for patients with clear margins; however, outcomes with the two different regimens were similar (9). In a recent retrospective analysis based on the National Cancer Database (NCDB), again both treatment provided similar 5-year OS with 67% after surgery-only and 65% after RT-only (10).

A meta-analysis by Morisod et al. included 729 patients with early-stage OPSCC (T1-2 N0 M0). The authors demonstrated a 5-year DSS of 90.4% in the RT group vs. 89.6% in the TOS group, suggesting TOS to be equally effective in terms of tumor control (11).

## Functional Outcome of OPSCC Patients

Recently, the results from the ORATOR trial (NCT01590355) have been published, the first and only randomized comparison of RT vs. TORS plus neck dissection for patients with OPSCC (12). This trial was a multicenter, phase II randomized study comparing primary RT (with or without chemotherapy) and TORS with neck dissection (with or without adjuvant therapy) in patients with T1 or T2, N0-N2 OPSCC. Patients were stratified by p16 status. Primary endpoint was swallowing-related QOL at 1 year using the M.D. Anderson dysphagia index (MDADI). Sixty-eight patients were enrolled in this trial between 2012 and 2017. After a median follow-up of 27 months, total mean MDADI scores were higher in the RT arm in comparison to TORS-based treatment [86.9 (SD 11.4) vs. 80.1 (SD 13.0)]. Despite this, the difference did not meet the pre-specified threshold (10 points) for a clinically meaningful difference. Worth mentioning, in the TORS plus neck dissection group, final staging was pT2 in 15 patients, pT3 in 4 patients, and pN2 in 17 patients. In total, only 10 of 34 patients (29%) received surgery alone. Moreover, tracheostomies were recommended. Two-thirds of the patients received adjuvant treatment [24 of 34 (71%)]. Despite all this, the TORS arm performed significantly poorer and even the *post-hoc* analysis showed no difference between surgery-only and surgery with adjuvant treatment consolidating the concern for TORS as the primary treatment of this disease.

A cross-sectional study in locally advanced OPSCC compared side effects of surgery plus post-operative RT vs. concurrent chemoradiotherapy (CRT) based on the EORTC-QLQ-H&N35 questionnaire. The study group consisted of 57 patients. The authors demonstrated that surgical patients described more problems with swallowing (*p* = 0.042), social eating (*p* = 0.038), and social contacts (*p* = 0.0002). Instead, patients treated with concurrent CRT had more problems with teeth (*p* = 0.049), dry mouth (*p* = 0.022), and sticky saliva (*p* = 0.044) (13). It is to be speculated that one of the reasons for poor swallowing recovery in the surgery group was the number of open trans-cervical or combined transoral/trans-cervical procedures.

## Functional Outcome After RT

Xerostomia and dysphagia remain the most important side effects following RT in HNC patients (14). In a UK prospective cohort study of 167 patients treated with 3D-(C)RT, QOL and dysphagia were evaluated with the MDADI and UWQOL (University of Washington Head–Neck Quality of Life) questionnaires at 3, 6, and 12 months: All patients received three-dimensional conformal treatment. There was a worsening of patient-reported swallowing outcome by 18% from baseline at 3 months (mean difference in the MDADI score = 14.5; *p* < 0.001). More important, this worsening exceeded the minimally important clinical difference (MICD) of 10 points, indicating a clinically relevant worsening of swallowing for the patient. Furthermore, little improvement could be observed from 3 to 12 months. Another important finding was that dysphagia was significantly less pronounced in patients treated with 50 Gy compared to 63 Gy, confirming the notion that not only volume but also total dose plays a major role in swallowing dysfunction. In addition, patients identified dysphagia and reduced/sticky saliva in the UWQOL domains as the most devastating impairments. In particular, dysphagia was highly correlated with long-term functional and QOL outcomes (15).

Intensity modulated radiotherapy (IMRT) represents one of the most important technology-driven advances in modern oncology (16). Furthermore, no other group of cancer patients has gained more by the introduction of IMRT, in terms of reduction of long-term morbidity, than HNSCC patients. This can, to some degree, be explained by the high number of organs at risk in the head and neck region. Globally, IMRT allows delivery of the necessary dose to the tumor in a much more conformal way than was previously possible, by dividing the beam into multiple small volumes (beamlets) of varying intensity (17).

In a randomized controlled trial, 94 patients with OPSCC or hypopharyngeal cancer were treated between 2003 and 2007 with either 3D conformal RT (3D-CRT) or IMRT in the primary setting. The primary endpoint was the proportion of patients with xerostomia assessed by the LENT SOMA subjective side-effect scale 1 year after treatment. The results showed a significant reduction of xerostomia in the IMRT group, from 74% at 12 months in the 3D-CRT group to 38% in the IMRT group. Furthermore, non-stimulated saliva flow from the contralateral parotid was noted in 47% in the IMRT group compared to none of the 25 patients in the 3DCRT group (*p* < 0.0001) (18). These results have been underscored by the RTOG 0022 trial data, a phase I/II study in patients with early-stage OPSCC treated with moderately accelerated hypo-fractionated IMRT only: Patients achieved high tumor control rates [2-year estimated loco-regional-failure (LRF) rate 9%] and reduced salivary toxicity compared with similar patients in previous studies (19).

Multiple studies have specifically evaluated the potential benefit of IMRT in terms of functional outcomes and in particular of swallowing function. A table summarizes a selection of publications evaluating the impact of different RT techniques on dysphagia (Table 1).

**Table 1:** Selected studies on dysphagia outcomes following primary radiotherapy with IMRT.

**References**	**Technique**	**Combined treatments (%)**	**Stage (%)**	**Study type**	**No. patients**	**FU (months)**	**Dysphagia outcomes**
Anand et al. (20)	IMRT	RT alone: 29 CRT: 47 PORT:27	Stage III-IV: 77.3	Pooled analysis	62	19	**Chronic dysphagia based on the CTC grading Version 3.0 at 6 months** Grade I: 10.5% Grade II: 12.3% No Grade III
Feng et al. (21)	SW IMRT	CRT: 100	Stage III-IV: 100	Prospective cohort study	73	36	UWQOL^*^ & HNQOL^*^ At baseline: 10 & 8, at 24 months: 22 & 17
Schwartz et al. (22)	Split-field IMRT with laryngeal shielding	Weekly induction CT followed by CRT in 42 RT alone: 58	Stage IV: 100	Phase II trial	48	24	**MDADI** At baseline: 89.1 at 24 months: 82.4 No difference between CRT and RT-only treated patients.
Nutting et al. (18)	IMRT vs. 3D-CRT	RT alone: 68 or 83 PORT: 17 or 32 NeoadjCT:40 or 43	Stage III-IV-68 or 83	Phase III RCT	94	44	**LENT SOMA late side-effects: Dysphagia grade** **≥3** **At 12 months:** IMRT: 9% 3D CRT: 5% At 24 months, no difference between non-xerostomia late toxicities between two groups
Roe et al. (23)	PS IMRT	RT alone: 21 CRT: 7 Induction CT + CRT: 72	Stage III-IV: 92	Pooled analysis	62	12	**MDADI** At baseline: 86.9 At 3 months: 66.1 At 12 months: 71.3
Mazzola et al. (24)	IMRT	CRT: 54% PORT: 29%	Stage III-IV: 68	Pooled analysis	56	12	**EORTC/RTOG scoring criteria Chronic dysphagia** at 12 months Grade I: 91% Grade II: 9%
Goepfert et al. (25)	Split-field IMRT with laryngeal shielding	Induction CT. +concurrent CT: 28% Induction CT: 43% Concurrent CT: 57%	Stage III-IV: 100%	Pooled analysis	46	24	**MDADI** At baseline: 90 At 6 months: 74.6 At 12 months: 78.5 At 24 months: 83.1 15% had a depressed score by at least 20 points at 24 month
MD Anderson Head and Neck Cancer Symptom Working Group (26)	Split-field IMRT/whole field	CRT	T1-T4 N0 M0	Pooled analysis	300	48	**Chronic RAD:** 11%

*PS, Parotid-sparing; SW, Swallowing-sparing; CT, Chemotherapy; CRT, combined chemoradiotherapy; RAD, radiation induced dysphagia*.

* *Scores are in a scale of 0 to 100; higher scores denote worse symptoms*.

In addition to IMRT, new concepts have emerged to further actively spare swallowing and salivary organs with the aim of preserving swallowing function.

A prospective study by the Michigan group evaluated the additional benefit of improved sparing of swallowing structures such as the pharyngeal constrictors, the glottic and supraglottic larynx in patients with stage III and IV OPSCC treated with primary chemo-IMRT: In order to achieve this, the authors defined clear dosimetric goals for these structures (*D*_max_ < 50 Gy). Additionally, the medial retropharyngeal nodes were not included in the elective target volumes and the additional margin from the microscopic extension to the planning target volume was minimized (0.3 cm) (27): Overall, 73 patients were treated with a median follow-up of 36 months. The primary endpoint was patient-reported dysphagia assessed by the Eating Domain of the Head and Neck Quality of Life questionnaire (HNQOL) and the UWQOL. At 1 year, observer-rated dysphagia was absent or minimal; however, patient-reported scores worsened by 10 (UWQOL) and 13 (HNQOL) points, respectively. Three-year DFS was 88%, which is similar to standard IMRT treatments (21).

Pooled data from two prospective cohort studies, with a median follow-up of 6.5 years, in which patients were treated with swallowing and salivary organ-sparing IMRT, reported stable or improved HRQOL in comparison to prior treatment (28).

Despite the nowadays standard use of IMRT in this patient group, RT still has a significant impact on swallowing-related QOL parameters: In a prospective cohort study of 62 patients diagnosed with HNSCC treated with IMRT or chemo-IMRT, Roe et al. showed a decrease of 20.8 points in swallowing performance using the MDADI from baseline to 3 months after treatment. A statistically significant improvement could be observed between 3 and 12 months (15.7 points from baseline, *p* = 0.04) (23).

In another retrospective trial of highly selected patients with low to intermediate risk OPSCC who were treated with laryngeal/esophageal inlet dose-optimized IMRT, a decrease of 15 points in the MDADI scale was observed at 6 months. A statistically significant improvement was noticed at 24 months (*p* = 0.02) in comparison to baseline. This suggested that patients with OPSCC treated with esophageal inlet dose optimized IMRT are highly likely to report recovery of acceptable swallowing function on long-term follow-up (25).

Finally, the MD Anderson Head and Neck Cancer Symptom Working Group analyzed, in a cohort of 300 patients treated with CRT between 2002 and 2011, the rate of chronic radiation-induced dysphagia (RAD) at >12 months post-IMRT. RAD was defined as (1) aspiration/stricture at video-fluoroscopy/endoscopy, (2) gastrostomy tube, and/or (3) aspiration pneumonia. In total, they found a rate of 11% chronic RAD at 12 months. Interestingly, age and volume receiving more than 69 Gy were the most predictive covariates.

## Functional Outcome After Transoral Surgery

Minimally invasive techniques, such as transoral laser microsurgery (TLM) and TORS, have emerged within the past two decades. The major aim of these novel strategies is to reduce treatment-related morbidity while preserving excellent oncological control. Swallowing function has been evaluated after TLM and TORS in several studies (Table 2) and overall outcomes have been promising.

**Table 2:** Dysphagia outcomes of trans-oral surgical (TOS) approaches for oropharyngeal squamous cell carcinoma (OPSCC).

**References**	**Surgical procedure**	**Stage (%)**	**Combined treatments (%)**	**Study type**	**No. patients**	**FU (months)**	**Dysphagia outcomes**
Iseli et al. (29)	TORS	T1:35, T2:44 Stage III-IV: n/a	Neoadjuvant RT:22 PORT:41 POCRT: 31	Prospective cohort	54	13	Mean MDADI score declined from 75 to 65 at 2 months
Moore et al. (30)	TORS	T1:33, T2:40 Stage III-IV: 86	PORT: 18 POCRT: 56	Prospective case study	45	12.3	**FOSS score** In case of TORS only, FOSS score returned to 0 at 4 weeks
Genden et al. (31)	TORS	T1:53, T2:43 Stage III-IV:73	PORT: 10 POCRT: 27	Prospective case control study	30	14.8	FOIS score^*^ returned to baseline at 12 months in the TORS-only group
Sinclair et al. (32)	TORS	T1:45, T2:55 Stage III: 76 No Stage IV	PORT: 45 CRT: 31	Prospective case series	42	17	**MDADI score** At baseline: 82 After median FU:74
Moore et al. (33)	TORS	T1:46, T2:39 Stage III-IV: 90	PORT: 21 POCRT: 62	Prospective study	66	34	95.5% of patients maintain nutrition without feeding tube
More et al. (34)	TORS	T1:30, T2:35 Stage III-IV:100	PORT: 40 POCRT:60	Prospective comparative study	20	14	**MDADI score** returned to baseline (78 points) in the TORS group
Chen et al. (35)	TORS/TLM	T1:52, T2:39 83>N1	PORT: 84 POCRT: 16	Retrospective matched pair analysis	31	20	**UW-QOL sore** at 1 year 91.5 vs. 72.1 in patients treated with TOS +adj. treatment
Sethia et al. (36)	TORS	T1: 62 T2:38 46 > N1	TORS alone:12 PORT: 28 POCRT:60	Prospective cohort study	111	35	**HNCI eating domain score** for TORS alone was higher than for adjuvant (C)RT at 3 months. (*p* < 0.01)
Morisod et al. (37)	TORS	19 T1/T2N0 10 T1/T3N1-2B Stage III-IV: 31	PORT: 28 CRT: 3	Prospective cohort study	29	20	In the TORS only group: 67% with stable or improved FOSS score

* *Mean FOIS: Mean Functional Oral Intake Score, PORT: Post-operative RT; POCRT: Post-operative CRT; TLM: Trans-oral laser-microsurgery*.

A prospective study from the Mayo Clinic recruited 45 patients with OPSCC for TORS-based treatment. Swallowing function was assessed by the Functional Outcome Swallowing Scale (FOSS), an observer rated dysphagia outcome scale: All patients who were treated with TORS and neck dissection only and with a baseline score of 0 had a FOSS score of 0 at 4 weeks after surgery, whereas patients after adjuvant CRT were found to have FOSS scores of more than 2 at 3 months after finishing treatment (30).

Another prospective case–control study was done by the Mount Sinai School of Medicine, New York: They included 30 patients with mostly stage III–IV OPSCC (7th AJCC classification) treated with TORS and adjuvant therapy. Subjective and objective functional outcomes were measured with the Performance Status Scale for Head and Neck Patients (PSS-HN) and the Functional Oral Intake Score (FOIS). After a median follow up of 14.8 months, they showed better functional outcome with less eating- and diet-related changes in comparison to controls treated with definitive CRT at 2 weeks (5.5 vs. 3.3; *p* < 0.001). However, at 3, 6, 9, and 12 months, there were no significant differences (31).

Sinclair et al. included 42 patients with T1 or T2 OPSCC in a study aiming to evaluate based on the MDADI score patient perceived swallowing function after TORS +/– RT or CRT: After a median follow-up of 13.6 months, the mean MDADI score after TORS was 74.3, which was comparable to that of patients treated with CRT in other studies (23, 32, 38).

The group from Kansas led a prospective non-randomized trial in patients with stage III or IV (7th AJCC classification) OPSCC and SGSCC treated by either TORS with adjuvant RT or definitive CRT. Primary endpoint was swallowing function based on the MDADI score. At a mean follow-up of 14 months, they found a significantly better swallowing MDADI outcome compared to primary CRT at 12 months (78 vs. 60; *p* = 0.006) with a return to baseline in the surgery arm. In comparison, in the CRT group, a gradual improvement to 60 was made, but no return to baseline (34).

In another study by Chen et al. a matched pair analysis in patients with stage I–IV tonsils and base of tongue tumors (7th AJCC classification) was performed. They evaluated functional outcomes after TOS followed by risk-stratified adjuvant treatment and primary CRT. Sixteen patients were treated with TLM and 15 patients were treated with TORS. The endpoint was QOL at 1 year based on the UWQOL score: Subjective reporting of swallowing was better in the TOS with adjuvant RT group in comparison to the CRT group at 1 year (91.5 vs. 72.1; *p* = 0.01) (35).

The head and neck group from Ohio, USA, evaluated the impact of adjuvant treatment after TORS on QOL in 111 patients treated for OPSCC from 2005 to 2015. With a mean follow-up of 35 months, based on the Head and Neck Cancer Inventory (HNCI), TORS alone reported significantly higher eating scores than after adjuvant RT or CRT at 3 and 6 months (*p* < 0.01) (36).

In a single institutional prospective cohort study on patients with T1-2 N0-2b OPSCC, of which approximately 50% were treated for secondary oropharyngeal primaries, the percentage of patients with low FOSS scores (FOSS 0-2) did not significantly change after TORS-based treatment (89 vs. 88.5%), indicating low dysphagia rates after TORS, even in patients with previous HNC treatments (37).

Finally, a systematic review of 11 studies (190 patients) in early-stage OPSCC treated with TORS revealed a 5% incidence of late gastrostomy tube dependence at more than 12 months follow-up. In most studies, a near-complete recovery was documented with changes smaller than the “minimally important clinical difference” (7, 39).

## Design of the EORTC 1420 “Best of” Trial

The EORTC 1420 “Best of” trial is an ongoing open-label phase III prospective randomized trial assessing the “best of” surgery compared to the “best of” RT initially in patients with T1-2 N0 OPSCCs. This was recently amended to also include T1-2 N0-1 OPSCC, SGSCC, and T1 N0 hypopharyngeal squamous cell carcinoma that are p16 positive and p16 negative. The main objective of the study is to assess and compare patient-reported swallowing function based on the validated MDADI composite score over the first year after randomization between TOS and IMRT (40, 41). Key secondary endpoints are common oncological endpoints as well as functional and HRQOL measures based on the PSS-HN, 100 ml swallow test, feeding tube use, and the EORTC QLQ-C30 and HN43.

Besides this main objective related to patient-reported swallowing outcome comparing two treatment techniques, a second objective is to identify the most optimal treatment for early-stage HNSCCs in general that are amenable to single-modality treatment. This certainly excludes tumor sites where no controversy exists (i.e., oral cavity, nasopharynx). Finally, EORTC 1420 is equipped with a translational research program that attempts to run large-scale proteomics analysis on the saliva of patients, in order to develop biomarkers allowing for the prediction of functional recovery after one or the other treatment. This objective may even be the most important given that being able to predict functional recovery after various treatments based on a biomarker prior treatment would solve the entire controversy.

A total number of 170 patients (85 patients in each arm) will be recruited to this study. Stratification factors are tumor localization, N-stage, MDADI at baseline, and country. After a 1:1 randomization into TOS or IMRT, patients are followed at four separate time points for patient-reported swallowing outcome in order to assess the evolution of swallowing recovery with each treatment technique.

In the TOS arm, any transoral technique (i.e., TLM, TORS, etc.) can be used. Of importance is a quality assurance program allowing for harmonization of techniques at a very high quality level (42). In order to avoid post-operative RT in the surgery arm, a re-resection policy was added to the protocol, requiring the surgeon to revise a positive or close margin unless refused by the patient. Also, tracheostomies are not in general recommended, helping with the recovery of swallowing. In comparison to the ORATOR protocol, the surgical procedures outlined in the EORTC 1420 protocol are less invasive and allow other well-established techniques such as TLM also to be performed.

For the IMRT arm, clinical target volumes for the primary tumor and nodes as well as the swallowing structures are contoured according to international guidelines (43, 44) and strict dose constraints are defined concerning relevant swallowing structures based on current literature (22, 26, 27, 45, 46). Additionally, in order to reduce protocol deviations, an extensive patient-specific quality assurance program was implemented.

## Discussion

Patients recruited for EORTC 1420 “Best-of” will be randomized between two currently “state of the art” treatments in their respective field. The TOS arm offers a way of reducing toxicity by accessing the tumor through the mouth and removing the disease via minimally invasive techniques using either a robotic system or a laser. Clearly defined quality assurance guidelines and an accreditation process for surgeons assure state-of-the-art conduct of the operations and comparability of interventions (42).

The RT arm not only consists of a mandatory IMRT technique with reducing dose to the contralateral parotid gland, as it is used currently in most hospitals today, but also contains defined dose constraints to different swallowing organs that are needed to be contoured separately. Furthermore, a strict quality-assurance program has been put in place in order to guarantee optimal execution as it has been shown that deviation from clinical protocol can have an impact on clinical outcome (47).

As reported, the recently published ORATOR trial was a phase II study, which compared RT-based and TORS-based treatment in OPSCC in terms of swallowing function at 1 year. Notwithstanding the similarities between BEST-OF and ORATOR in light of study design, patient selection, and primary endpoint, some differences need to be mentioned:

1. In BEST-OF, not only OPSCC tumors are included, but also supraglottic and T1 N0 hypopharyngeal carcinomas. In addition, patients are stratified according to tumor localization. It was also one of the conclusions from the ORATOR trial that maybe certain subsites (tonsil or base of tongue) or T-stages could help select the best treatment option in the future.
2. Only N1 disease is allowed in BEST-OF as compared to a maximum lymph node dimension of 4 cm in any plane, on either side of the neck in the ORATOR trial (N2 disease). This is potentially relevant as in ORATOR, 72% (*n* = 23) received concurrent CRT in the RT arm and 24% (*n* = 8) adjuvant CRT in the TORS arm.
3. In ORATOR, only TORS was allowed; however, in BEST-OF, TLM and conventional transoral surgery are also permitted.
4. BEST-OF runs with an integrated quality program for surgery consisting of a credentialing part and the follow-up on various process and outcome indicators. The procedures in BEST-OF seem less invasive and more consistent with current European standards of transoral organ preservation surgery, i.e., no mandatory tracheostomy, possibility of margin revision, and precautions to avoid resecting T3-disease.

In conclusion, BEST-OF is a phase III randomized trial, which compares two different techniques (IMRT vs. TOS) in earlier stages for multiple subsites as opposed to ORATOR, a phase II trial, which compared two different multi-disciplinary treatment strategies in more advanced disease and only for OPSCC.

Despite an abundance of published literature on acute and late dysphagia after RT- or TOS-based treatments, it is challenging to make unequivocal conclusions as to what treatment should be prioritized, due to a high number of methodological variations and the non-randomized nature of most comparisons.

In addition, due to the high complexity of the impairment, many cofounders exist: age, dental status, pretreatment dysphagia, gender, location, and tumor volume, to name a few (48). Since both novel surgical and radiation techniques appear to offer equivalent efficacy in terms of tumor control, it is essential to evaluate which one would be superior in preserving function. Therefore, only a randomized phase III design can elucidate which treatment is best in which situation by properly evaluating the respective benefits and disadvantages of these treatments and highlight which one of the modalities will provide better functional outcome.

Surgery and RT still represent the backbone of curative treatment for OPSCC (49). It would be ideal if such interventions would come with limited or no morbidity, so that patients, if requiring multi-modality treatment, could either move on to adjuvant or receive additional treatments without any sequels from their primary treatment. Therefore, the results from this study are of major value as they help to define this backbone of treatment that should be used in the future in combination with additional, i.e., systemic therapies in advanced disease or novel de-intensification regimens.

## Conclusion

The question addressed in “BEST-OF” is timely and of interest to the community as it responds to a major public health problem. Dysphagia is a key symptom related to many comorbidities like poor nutrition, dehydration, aspiration pneumonia, feeding tube dependence, depression, and isolation (50, 51). Chronic aspiration is a particularly serious and often underestimated side effect among OPSCC patients undergoing CRT, with a reported rate of 7.6% (52). Within a growing and aging world population with an increase of human papillomavirus-driven pharyngeal tumors, dysphagia will become of even more importance in the coming years. Therefore, this trial still responds to an urgent unmet need to devise efficient dysphagia-sparing treatment strategies in a randomized and pragmatic design (2, 53, 54).

## Author Contributions

CS and J-JS: conceptualization, methodology, data acquisition, and supervision. CS, J-JS, VG, VP, WG, MS, TJ, MA, MB, RG, JK, ME, JB, CL, GS, AD, KH, FZ, IT, JP, SQ, A-SG and CF: writing.

### Conflict of Interest

The authors declare that the research was conducted in the absence of any commercial or financial relationships that could be construed as a potential conflict of interest.
